# General regression methods for respondent-driven sampling data

**DOI:** 10.1177/09622802211032713

**Published:** 2021-07-28

**Authors:** Mamadou Yauck, Erica EM Moodie, Herak Apelian, Alain Fourmigue, Daniel Grace, Trevor Hart, Gilles Lambert, Joseph Cox

**Affiliations:** 1 Department of Epidemiology, Biostatistics an Occupational Health, McGill University, Montreal, Quéebec, Canada; 2 Dalla Lana School of Public Health, University of Toronto, Toronto, Ontario, Canada; 3 Department of Psychology, Ryerson University, Toronto, Ontario, Canada; 4 Institut National de Santé Publique du Québec, Montreal, Québec, Canada

**Keywords:** Design weights, hidden population sampling, homophily, identifiation, peer effects, simultaneous autoregressive models, social networks

## Abstract

Respondent-driven sampling is a variant of link-tracing sampling techniques that aim to recruit hard-to-reach populations by leveraging individuals’ social relationships. As such, a respondent-driven sample has a graphical component which represents a partially observed network of unknown structure. Moreover, it is common to observe *homophily*, or the tendency to form connections with individuals who share similar traits. Currently, there is a lack of principled guidance on multivariate modelling strategies for respondent-driven sampling to address peer effects driven by homophily and the dependence between observations within the network. In this work, we propose a methodology for general regression techniques using respondent-driven sampling data. This is used to study the socio-demographic predictors of HIV treatment optimism (about the value of antiretroviral therapy) among gay, bisexual and other men who have sex with men, recruited into a respondent-driven sampling study in Montreal, Canada.

## 1 Introduction

*Respondent-driven sampling* (RDS) is a network-based sampling technique that leverages social relationships to recruit individuals of hard-to-reach populations into research studies.^1^ The RDS process, which proceeds through recruitment *waves*, starts with the selection of initial *seed* participants who, after being interviewed, receive a fixed number of *coupons* to distribute among their peers. RDS offers many advantages over existing network-based sampling methods. Through many waves of recruitment, the process samples farther from the initial recruits, which should ensure greater representativeness and hence generalizability of the sample. This is because seeds typically represent a convenience sample, even if thoughtfully chosen with the view to optimizing representation of their social spheres. Moreover, RDS reduces the privacy concerns that are associated with the identification of participants’ social networks or the community population that could occur in a more traditional study that would aim to enumerate the members of the target population by relying on members to recruit their peers into the study.

An RDS sample has a graphical structure, which is typically a partially observed social network of recruited individuals with an unknown underlying dependence structure in which it is common to observe a tendency for individuals with similar traits to share social ties, a feature termed *homophily*. Moreover, the RDS process is not one that is purely random, but rather some individuals are more likely to be selected into the sample than others. An assumed underlying principle in RDS is that the probability of an individual being recruited depends on the size of their personal network of social contacts.^1,2^ However, the true RDS sampling design is unknown, warranting inferential methods that rely on approximations to the true RDS process to estimate design weights.

The current literature of RDS data lacks principled guidance on multivariable modelling.^3^ This is reflected in the variety of analytic approaches taken in the applied literature. Some studies have treated RDS data as though collected by random sampling and applied analysis of variance, linear and logistic regressions without any adjustment for RDS weights.^4^ Others have included RDS weights in regression models, relying on the typical RDS assumption that some individuals are more likely to be recruited into the sample than others, while ignoring the dependence between observations within the RDS network.^5^ Some researchers proposed including seeds as random effects to adjust for the dependence within recruitment chains but ignored RDS weights.^6^ A mixed effects model including random effects on features such as seeds and recruiters to account for the dependence, using weights at different levels of clustering when appropriate, and modelling homophily-driven effects by including a parameter to account for possible interactions between recruiters and recruits’ values of homophilic covariates has been proposed.^7^ This approach was presented as a general guidance for RDS regression; however, no theoretical details or practical (simulation) demonstrations of the performance of the proposed methodology were provided.

Thus, while there are well-developed strategies for estimating means and prevalences from RDS studies, best practices for regression modelling remain poorly characterized. And yet, understanding dependence between variables is often a primary goal in epidemiologic research. Take for example the question of whether socio-demographic characteristics can predict optimism about the value of antiretroviral therapy, either as a pre-exposure prophylaxis or post-infection treatment, in a population of gay, bisexual and other men who have sex with men (GBM). There have been suggestions that younger people (aged less than 35) were less likely to have optimism, while people with lower annual income (less than $20,000) were more likely to have optimism,^8,9^ which could potentially mitigate the effectiveness of HIV preventive measures in some subgroups of the GBM population. The Engage study, which is an RDS study conducted in Montreal, Toronto and Vancouver, provides a unique opportunity to study this question in a large sample of the GBM community – but doing so requires appropriate modelling strategies.

One of the most challenging issues of multivariate modelling for RDS is one of missing data. In fact, the observed data reveal partial information about the full RDS network in which all connections between recruited individuals are reported.^10,11^ This problem is fundamentally design-based.^12^ A critical question is how this missing information and concerns regarding the identifiability of network parameters impacts the estimation of *regression* parameters associating variables measured on individuals in partially observed networks.

The paper is organized as follows. In Section 2, we provide a brief background to respondent-driven sampling and define the resulting network structure of an RDS sample where social connections can be viewed as exhibiting a correlation structure that is analogous to a spatial pattern (where the ‘distance’ metric is the number of social separations between individuals). In Section 3, we propose a generalized mixed effects model, with peer effects driven by homophily and with spatial random effects to model the dependence between outcomes within the network. We briefly discuss the issue of statistical inference when the full network of recruited individuals is only partially observed by design, and the inclusion of RDS weights to account for the non-random sampling of the target population when recruited individuals (accurately) report on their personal network sizes. The validity of the proposed methodology is investigated in simulations presented in Section 4. In Section 5, we analyse the Engage data collected in Montreal to investigate the relationship between HIV treatment optimism and socio-demographic characteristics, providing reliable parameter estimates and appropriate standard errors via our proposed approach. We conclude in Section 6 with a discussion of the approach and future considerations.

## 2 A brief review of RDS

In this section, we briefly review the assumptions needed for an RDS design, and graphically display an example of the resulting observed network structure – which is a partial view of the underlying network structure.

Suppose an infinite population in which individuals are connected by social ties. We define this as the population network and state the following:

**Assumption 1** (**The population network**). The population network represents an infinite number of non-overlapping clusters of finite sizes.

In other words, the population is clustered, with individuals partitioned into well-defined clusters. Note that in much of the RDS literature, the population is assumed to form one connected network. We believe that to be an overly restrictive and unrealistic assumption. For example, the Colorado Springs Project 90 study^13^ revealed a real-world social network of 125 disjoint clusters.

Now, consider an RDS process operating across social connections of the population network.

**Assumption 2** (**The RDS recruitment**). *The recruitment process takes place within a subset of clusters of the network and progresses across individuals’ social connections*.

This assumption implies that the RDS sampling process can be characterized as a two-stage sampling design in which seeds and then, subsequently, additional individuals are selected from non-overlapping clusters. Note that this assumption has no implications on inferential approaches for means and proportions.

**Assumption 3** (**No multiple recruitments**). *No individual can be recruited more than once into the study*.

This assumption has been made in previous work on theory for RDS estimators of means.^2^ The above three assumptions imply that the observed RDS network can be represented as a finite set of non-overlapping trees. For practical purposes, consider the Engage study in Montreal. The RDS recruitment consisted of three main steps.

Step 1. Sampling started off with the purposeful selection of a first group of 27 GBM, the seed participants. Seeds were selected to be representative with respect to the diversity of the GBM community based on a community mapping exercise. The seeds were invited to a community-based survey site to complete a questionnaire and to undergo testing for sexually transmitted and bloodborne infections. Seeds who successfully completed the study received a (monetary) remuneration known as a primary incentive. This is wave zero of recruitment.

Step 2. All seed participants were each given six uniquely identified coupons and asked to recruit their GBM peers into the study; the social ties between a recruiter and any new participants recruited were then known to the study through the coupon and recorded in the study database. Successful recruiters received a secondary (monetary) incentive for each peer that they recruited.

Step 3. The process continued through successive waves until the desired sample size was reached.

## 3 Methodology

In this section, we jointly model homophily-driven effects and the dependence between outcomes from the clusters of the unobserved population network. This allows us to view the fitting of the assumed model to the observed RDS data as a missing data problem. The resulting identification issue is discussed in Section 3.2. Common strategies to account for the non-random sampling of the population and the question of whether to weight the model are discussed in Section 3.3.

### 3.1 Underlying, data-generating model and assumptions

Let *y_ij_* be the outcome measured on the *j*th individual of the *i*th cluster, $j=1,\ldots ,N_{i}$ , where *N_i_* is the size of the *i*th cluster, and i=1,…,m . Let *x_ij_* be the value of the covariate for the *j*th individual of the *i*th cluster, and $x_{i}$ the vector of covariates for all individuals in the *i*th cluster. We assume that $DP=\{y_{ij},x_{ij};i=1,\ldots ,m;j=1,\ldots ,N_{i}\}$ is the realization of a random sample whose distribution is identical to that of the superpopulation of clusters defined in Section 2, so that any inference based on the sample pertains to the parameters of the infinite population from which the sample is drawn. We assume that the underlying relationship between the outcome and covariates in the population is characterized by a generalized linear mixed model in which *δ_ij_* is the random effect for the *j*th individual of the *i*th cluster, $\mu_{ij}=\text{E}\left(y_{ij}|x_{i},\delta_{ij}\right)$ , and

(1) $$g\left(\mu_{ij}\right)=\beta_{0}+\beta_{1}x_{ij}+\gamma n_{ij}^{-1}\sum_{k∼j}x_{ik}+\delta_{ij}$$

where g(.) is a monotonic function of the mean, k∼j represents the set of individuals who share ties with the *j*th individual, *n_ij_* is the number of social connections that the *j*th individual of the *i*th cluster shares with other individuals within the same cluster, or *degree*. We further assume that $\delta_{i}=\left(\delta_{i1},\ldots ,\delta_{iN_{i}}\right)∼N(0,\Sigma_{i})$ , with $\text{cov}\left(\delta_{i},\delta_{j}\right)=0$ for i≠j . The parameter *γ* measures homophily-driven effects, or the influence of peerster, or l to that of the superpopulation of clust.^14^ In this model, the parameters *β*_0_ and (the potentially vector-valued parameter) *β*_1_ are of primary interest.

The form of $\Sigma_{i}$ is defined as follows. Let $S^{\left(i\right)}$ be a *neighbourhood* matrix representing social ties within the *i*th cluster, with elements ${\text{s}}_{jk}^{\left(i\right)}$ such that ${\text{s}}_{jk}^{\left(i\right)}=1$ if the *j*th and the *k*th individuals share a tie, ${\text{s}}_{jk}^{\left(i\right)}=0$ otherwise, ${\text{s}}_{jj}^{\left(i\right)}=0$ and $S=\text{diag}\{S^{\left(i\right)}\}$ . We assume a simultaneous autoregressive (SAR) model^15,16^ for the vector of random effects $\delta_{i}$

(2) $$\delta_{i}=\rho S^{\left(i\right)}\delta_{i}+u_{i}$$

where *ρ* represents the strength of the dependence within the network and $u_{i}∼N(0,\sigma^{2}I_{N_{i}})$ . Given $W_{i}={\left\{I_{N_{i}}-\rho S^{\left(i\right)}\right\}}^{-1}$ exists, the covariance of $\delta_{i},\;\Sigma_{i}$ , can be written as

(3) $$\Sigma_{i}(\sigma^{2},\rho )=\sigma^{2}W_{i}^{2}$$

The SAR correlation matrix is such that outcomes from *neighbouring* (i.e. socially connected) individuals are more correlated than outcomes from non-neighbours. Other correlation models for $\delta_{i}$ with such properties include conditional autoregressive (CAR) models, which belong in the same class of areal models as SAR models,^17^ and models which assume a correlation function that depends on a metric between observations.^18^

### 3.2 The validity of inference

Given {S,DP} , valid statistical inference for $\left(\beta_{0},\beta_{1},\gamma ,\sigma^{2},\rho \right)$ is straightforward. This section discusses the validity of classical inferential procedures when the population network is partially observed and proposes alternative model fitting strategies.

Consider the observed data from RDS $DT=(y_{ij},x_{ij};i=1,\ldots ,m,j=1,\ldots ,n_{i})$ , where *n_i_* is the number of recruits belonging in the *i*th cluster. Let $S_{T}$ represents the observed neighbourhood matrix for the RDS recruitment tree. When data are collected under traditional RDS designs, the complete information on recruited individuals {S,DP} is only partially observed through $\left\{S_{T},\;DT\right\}$ . In a more general network setting, Chandrasekhar and Jackson showed how using sampled network data leads to biases in regression parameters estimators.^19^ Thus, fitting model (1) to the observed RDS data $\left\{S_{T},\;DT\right\}$ might be an ineffective strategy.

First, consider the modelling of homophily-driven effects in (1). Under the assumption that $\text{plim}\sum_{j,k}\left(x_{ij}-{\bar{x}}_{i}\right)(x_{ik}-{\bar{x}}_{i})=0$ as n→∞ for all *i*, which implies that the covariate for *γ* is not correlated with that for *β*_1_, then valid inference for *β*_1_ is possible given the incomplete information $\left\{S_{T},\;DT\right\}$ . In this case, ignoring *γ* in the fitted model – which cannot be consistently estimated given the observed data, does not affect the validity of classical inferential methods for *β*_1_.

Furthermore, consider the SAR model (2) for the random effects $\delta_{i},\;i=1,\ldots ,m$ . For the aforementioned reasons, the parameter vector $\left(\sigma^{2},\rho \right)$ of the network-induced correlation structure (3), which is a function of the neighbourhood matrix of social ties, cannot be consistently estimated given $\left\{S_{T},\;DT\right\}$ . Other network-induced correlation structures such as the autoregressive, the ‘RDS-tree’^20^ and the Toepliz, although suitable for the branching structure of the recruitment tree, also fail to adequately capture the network-induced correlation structure, and/or are simply inestimable, for the same reasons.

In light of these results, and for the purpose of conducting valid inference for *β*_1_, we propose a model fitting strategy in which the homophily-driven effects are ignored. To capture the dependence between observations within trees, we consider an alternative class of correlation models for which the dependence within the *i*th tree is induced by a cluster-specific random effect $\delta_{i}=\delta_{i},\;i=1,\ldots ,m$ ; clustering is assumed at the seed level and at the recruiter level.^7^ The finite sample performance of our methodology for linear, Poisson and logistic regressions, in terms of accuracy and precision for the maximum likelihood estimator (MLE) of *β*_1_, and the coverage of the 95% confidence interval for *β*_1_, in these cases of omission of a non-confounding covariate and model misspecification for the random effects, will be investigated via simulations in Section 4.

### 3.3 RDS weights

When conventional sampling methods are used to gather information on a target population, sampling probabilities are known throughout the sampling process. This allows the researcher to compute and take into account design weights when estimating finite population parameters. These approaches are infeasible in an RDS setting since sampling probabilities are unknown. The sampling process is only (partially) controlled by the researcher through the selection of an initial set of seeds – who, while carefully chosen, still represent a convenience sample – with the remainder of the recruitment working through a sampling mechanism that relies on individuals’ social networks and personal decisions. Let *R_ij_* = 1 if the *j*th individual in the *i*th cluster is sampled. If the true sampling design S were known, the inclusion probability of the *j*th individual in the *i*th cluster would be computed as

$$\pi_{ij}=\text{E}(R_{ij}|S)$$

The RDS process can be approximated as a random walk on the nodes of an undirected graph,^21^ and RDS samples can then be treated as independent draws from its stationary distribution. The resulting inclusion probability for the *j*th individual is estimated by

$${\hat{\pi }}_{ij}^{\mathrm{RDS}-II}=n_{ij}^{-1}n^{-1}\sum_{i=1}^{m}\sum_{j=1}^{n_{i}}n_{ij}$$

Recalling that *n_ij_* is the number of social connections that the *j*th individual of the *i*th cluster shares with others in the same cluster, these weights have the appealing intuition of adjusting for the ‘popularity’ of an individual, and hence their likelihood of being recruited. The resulting estimators for means and proportions can be severely biased when sample fractions are large, among other factors.^2^ They proposed successive sampling (SS) weights based on a SS approximation of the RDS sampling design, which is viewed as a probability proportional to size without replacement design, and showed that resulting estimators consistently outperform estimators based on RDS-II weights. They also provided details of the algorithm for computing the SS weights. An important drawback of this approach is that the computation of inclusion probabilities requires knowledge of the population size. Another is that the weights vary depending on the chosen outcome, and so must be computed anew for each outcome or analysis; this can be impractical in large, collaborative or multi-site studies.

Until recently, the majority of inferential methods in the RDS literature dealt with the estimation of population means or proportions. The use of RDS weights in these settings is principled and straightforward. The use of sampling weights in a regression setting is more challenging and has been widely discussed.^7,22^ In light of these discussions, we consider the use of unit-level weights dealt with the estimation of population means or proportions. The use of RDS weights in these settings is Printo account, as these are widely used in the RDS literature.

### 3.4 Bootstrap variance estimators

We consider two bootstrap methods for estimating uncertainty in RDS: (i) the *tree* bootstrap^23^ and (ii) the *neighbourhood* bootstrap.^24^

The tree bootstrap method is based on resampling the RDS tree. Bootstrap samples are typically drawn from the observed recruitment tree by mimicking its hierarchical structure. The first level of the tree generation consists of resampling with (or without) replacement from the sets of seeds of the observed recruitment tree. In the second level of the bootstrap procedure, we resample with (or without) replacement from each of the sampled seeds’ recruits. The third level is created by resampling from the wave 1 participants’ recruits. The process continues until there are no more recruits from which to sample. The tree bootstrap method mimics the recruitment tree and corresponding features such as the recruitment chain, the number of seeds and waves, thus taking into account the underlying network structure of RDS. Recent findings suggest that this method consistently outperforms existing bootstrap methods, but overestimates uncertainty.^3,23,24^

The neighbourhood bootstrap method^24^ is based on sequentially resampling individuals and their neighbours within the RDS tree. The first stage of resampling consists of uniformly selecting $n\times c_{r}^{-1}$ recruits, where *c_r_* is the average number of connections within the resampled RDS tree. We then include, in the second stage of resampling, the neighbours of all selected recruits in the bootstrap sample. This method captures the ‘local’ neighbourhood structure of the network by reporting all connections that a resampled unit has within the tree, without much reliance on its branching structure. The authors demonstrated its consistency and empirically showed that their method outperforms the tree bootstrap in terms of coverage, bias and mean interval width in a small sample setting.

## 4 Simulations

We conducted two separate simulation studies to assess the accuracy of regression parameter estimators under two distinct modelling scenarios. Under the assumption that equation (1) is the data-generating model, and that the variable *x* is uncorrelated with degree, the goal of the first simulation study is to assess the accuracy and precision for the MLE of *β*_1_, and the coverages of the 95% (model-based and bootstrap) confidence intervals for *β*_1_ if (i) homophily-driven effects *γ* are ignored when present and (ii) the correlation model (2) for the random effects is misspecified. We consider fitting the model without RDS weights, with RDS-II weights and with SS weights under three potential population sizes (one of which is correct). In the second simulation study, we assume a simpler version of the data-generating model (1) with no homophily-driven effects (implying that there are no missing covariates in the subsequent fitted model) and assess the performance of the MLE of *β*_1_ when the variable *x* is correlated with degree.

### 4.1 RDS sampling

We simulated networks using Exponential Random Graph Models,^25^ a class of generative models for modelling network dependence. Let **S** be the random adjacency matrix of the network, and **x** a vector of nodal attributes. The joint distribution of its elements is

(4) $$\text{P}\left(S=s|x,\eta \right)=\frac{\text{exp}\left\{\eta g\left(s,x\right)\right\}}{\kappa \left(\eta \right)}$$

where η is a vector of parameters and g(a,x) its corresponding vector of network statistics, $\kappa \left(\eta \right)=\sum_{s}\text{exp}\left\{\eta g\left(s,x\right)\right\}$ is a normalizing constant. The features of the network are captured in (4) by choosing network statistics to represent density (*d*) or the ratio of ties in the observed network over the total number of possible ties, degree distribution and homophily. The degree distribution is mainly controlled by setting different values for the geometrically-weighted degree parameter *η_G_* along with a ‘decay’ parameter *η_d_* that controls for the level of geometric weighting. When $\eta_{G}<0$ there are more high- and low-degree individuals than expected by chance, while when $\eta_{G}>0$ the network is more centralized.^26^ We simulated 10 clusters of equal sizes from which RDS samples were drawn for the following set of network characteristics: *N *=* *1000, d=1% and $\eta_{G}(\eta_{d})=-6(3)$ . We considered *s *=* *10 seeds, *c *=* *3 coupons and sample fractions of either f=20% or f=80% . We also considered RDS-II weights (*π_RDS_*), SS weights with *N* known (*π_SS_*), SS weights with ${\hat{N}}_{u}=N-(N-n)/2$ ( $\pi_{SS}^{u}$ ) and SS weights with ${\hat{N}}_{o}=N+(N-n)/2$ ( $\pi_{SS}^{o}$ ).

### 4.2 Regression models

In the first simulation study, we generated a continuous covariate *x* from a normal distribution with mean 3 and standard deviation 1.5. We define the following model

$$g(\mu_{ij})=\beta_{0}+\beta_{1}x_{ij}+\gamma n_{ij}^{-1}\sum_{k∼j}x_{ik}+\delta_{ij}$$

where *δ_ij_* follows the SAR model (3). We set the parameter vector to $\left(\beta_{0},\beta_{1},\gamma ,\sigma^{2})=(0,\;2,\;1.5,\;1\right)$ for each value of the autocorrelation parameter ρ=0.05, 0.1 . We considered three link functions: $g(\mu_{ij})=\mu_{ij},\;g(\mu_{ij})=\log(\mu_{ij})$ and $g(\mu_{ij})=\text{logit}(\mu_{ij})$ ; for the logistic model, we set the prevalence of the outcome variable to 30% by calibrating the intercept parameter to $\beta_{0}=-12$ using the cumulative distribution function of the logistic distribution. For each combination of network and sample characteristics, we fitted models in which the parameter *γ* is ignored.

In the second simulation study, we assume the following data-generating model

$$g(\mu_{ij})=\beta_{0}+\beta_{1}x_{ij}+\delta_{ij}$$

where *δ_ij_* follows the SAR model (3). The parameter vector is set to $\left(\beta_{0},\beta_{1},\sigma^{2},\rho )=(0,\;2,\;1,\;0.05\right)$ . We generated the continuous covariate *x* in such a way that the correlation with degree, measured using the Pearson correlation coefficient, is $\rho_{d}=0.4$ or 0.6. The setting for the link functions, the population network and the RDS process are identical to that of the first simulation study; the sample fraction is fixed at 20% across all combinations of simulation parameters.

To account for the dependence between observations in models for both simulations, we assumed clustering at both seed and recruiter levels, with seed-specific and recruiter-specific random effects. We weighted the models using the set of RDS weights described in Section 4.1; we assumed that each individual’s reported network size is precisely known. RDS-II and SS weights were computed via vh.weights and gile.ss.weights respectively, both functions of the R package RDS . We computed the relative bias and the root mean squared error of ${\hat{\beta }}_{1}$ , and the coverage of the 95% (model-based and bootstrap) confidence intervals for $\beta_{1}$ .

### 4.3 Results from the first simulation study: Ignoring homophily-driven effects and/or misspecifying the correlation model

Table 1 reports the relative bias and the root mean squared error of ${\hat{\beta }}_{1}$ , and the coverage of the 95% confidence interval for *β*_1_ in the linear, Poisson and logistic regression cases. Additional results for a smaller sample fraction ( f=10% ) are reported in Tables S1 to S3 of the Web supplement.

**Table 1.** Relative bias and root mean squared error of ${\hat{\beta }}_{1}$ , model-based CI, the TCI and the NCI for the 95% confidence interval of *β*_1_ for increasing levels of sample fraction (*f*), network dependence (*ρ*) and various RDS weights (*π*).

			f=20%					f=80%				
*ρ*	Clstr.	*π*	RB	RMSE	CI	TCI	NCI	RB	RMSE	CI	TCI	NCI
**Linear regression**												
0.05	S	1	0	0.06	0.96	0.99	0.94	0	0.03	0.94	1.00	0.92
		*π_RDS_*	0	0.07	0.90	0.98	0.93	0	0.05	0.82	1.00	0.92
		*π_SS_*	0	0.07	0.92	0.98	0.93	0	0.04	0.90	1.00	0.91
		$\pi_{SS}^{u}$	0	0.07	0.93	0.98	0.94	0	0.04	0.91	1.00	0.91
		$\pi_{SS}^{o}$	0	0.07	0.92	0.98	0.93	0	0.04	0.89	1.00	0.91
												
	R	1	0	0.07	0.95	0.99	0.94	0	0.03	0.94	1.00	0.95
		*π_RDS_*	0	0.08	0.88	0.99	0.93	0	0.05	0.81	1.00	0.95
		*π_SS_*	0	0.07	0.89	0.99	0.93	0	0.04	0.91	1.00	0.94
		$\pi_{SS}^{u}$	0	0.07	0.90	0.99	0.94	0	0.04	0.92	0.99	0.94
		$\pi_{SS}^{o}$	0	0.08	0.88	0.99	0.93	0	0.04	0.90	0.99	0.93
0.1	S	1	0	0.07	0.96	1.00	0.94	0	0.04	0.94	1.00	0.96
		*π_RDS_*	0	0.09	0.91	0.98	0.94	0	0.06	0.84	0.99	0.95
		*π_SS_*	0	0.08	0.94	0.99	0.95	0	0.04	0.93	0.99	0.95
		$\pi_{SS}^{u}$	0	0.08	0.94	0.99	0.95	0	0.04	0.93	1.00	0.95
		$\pi_{SS}^{o}$	0	0.08	0.93	0.99	0.94	0	0.04	0.92	0.99	0.96
												
	R	1	0	0.08	0.93	1.00	0.94	0	0.04	0.93	1.00	0.96
		*π_RDS_*	0	0.09	0.89	1.00	0.93	0	0.06	0.82	1.00	0.96
		*π_SS_*	0	0.09	0.89	1.00	0.92	0	0.04	0.91	1.00	0.95
		$\pi_{SS}^{u}$	0	0.09	0.91	1.00	0.92	0	0.04	0.91	1.00	0.95
		$\pi_{SS}^{o}$	0	0.09	0.89	1.00	0.93	0	0.04	0.91	1.00	0.96
**Poisson regression**												
0.05	S	1	−0.05	0.41	0.41	0.98	0.90	−0.08	0.30	0.29	0.87	0.80
		*π_RDS_*	−0.08	0.49	0.38	0.97	0.89	−0.12	0.38	0.23	0.82	0.79
		*π_SS_*	−0.08	0.48	0.39	0.97	0.89	−0.10	0.34	0.24	0.86	0.80
		$\pi_{SS}^{u}$	−0.08	0.46	0.38	0.98	0.89	−0.10	0.33	0.25	0.85	0.80
		$\pi_{SS}^{o}$	−0.08	0.48	0.40	0.97	0.89	−0.10	0.34	0.24	0.85	0.79
												
	R	1	−0.05	0.36	0.51	0.96	0.93	−0.06	0.24	0.36	0.97	0.92
		*π_RDS_*	−0.06	0.46	0.46	0.93	0.93	−0.09	0.30	0.31	0.96	0.94
		*π_SS_*	−0.08	0.47	0.53	0.94	0.93	−0.08	0.27	0.36	0.97	0.94
		$\pi_{SS}^{u}$	−0.08	0.45	0.52	0.95	0.93	−0.08	0.27	0.31	0.97	0.94
		$\pi_{SS}^{o}$	−0.08	0.47	0.55	0.93	0.92	−0.08	0.28	0.36	0.97	0.92
0.1	S	1	−0.08	0.43	0.42	0.92	0.90	−0.12	0.35	0.27	0.96	0.79
		*π_RDS_*	−0.10	0.44	0.37	0.90	0.88	−0.15	0.40	0.23	0.95	0.80
		*π_SS_*	−0.10	0.44	0.39	0.91	0.90	−0.14	0.37	0.24	0.95	0.79
		$\pi_{SS}^{u}$	−0.10	0.44	0.40	0.91	0.90	−0.13	0.37	0.24	0.95	0.78
		$\pi_{SS}^{o}$	−0.10	0.44	0.39	0.91	0.90	−0.14	0.38	0.24	0.95	0.80
												
	R	1	−0.10	0.52	0.47	0.97	0.96	−0.09	0.31	0.32	0.98	0.94
		*π_RDS_*	−0.11	0.51	0.48	0.95	0.96	−0.11	0.34	0.29	0.98	0.94
		*π_SS_*	−0.13	0.63	0.56	0.95	0.94	−0.10	0.33	0.32	0.99	0.94
		$\pi_{SS}^{u}$	−0.12	0.60	0.54	0.95	0.94	−0.10	0.33	0.33	0.99	0.96
		$\pi_{SS}^{o}$	−0.13	0.63	0.60	0.94	0.92	−0.10	0.34	0.33	1.00	0.96
**Logistic regression**												
0.05	S	1	−0.17	0.43	0.71	0.92	0.76	−0.21	0.43	0.43	0.59	0.45
		*π_RDS_*	−0.15	0.47	0.62	0.92	0.84	−0.23	0.49	0.19	0.73	0.42
		*π_SS_*	−0.16	0.46	0.63	0.93	0.82	−0.22	0.46	0.34	0.57	0.38
		$\pi_{SS}^{u}$	−0.16	0.46	0.64	0.92	0.80	−0.22	0.45	0.37	0.55	0.39
		$\pi_{SS}^{o}$	−0.16	0.47	0.63	0.93	0.83	−0.22	0.47	0.31	0.59	0.38
	R	1	0.12	1.35	0.63	1.00	0.98	0.01	0.43	0.54	1.00	0.98
		*π_RDS_*	0.15	1.19	0.63	1.00	0.99	0.03	0.36	0.61	1.00	0.97
		*π_SS_*	0.14	1.20	0.62	1.00	0.99	0.03	0.39	0.54	1.00	0.98
		$\pi_{SS}^{u}$	0.14	1.27	0.62	1.00	0.99	0.03	0.40	0.53	1.00	0.98
		$\pi_{SS}^{o}$	0.14	1.19	0.63	1.00	0.99	0.03	0.38	0.57	1.00	0.98
0.1	S	1	−0.24	0.53	0.61	0.85	0.74	−0.25	0.51	0.35	0.30	0.20
		*π_RDS_*	−0.21	0.52	0.53	0.95	0.82	−0.25	0.53	0.18	0.59	0.23
		*π_SS_*	−0.22	0.52	0.54	0.95	0.82	−0.25	0.52	0.30	0.32	0.25
		$\pi_{SS}^{u}$	−0.22	0.52	0.52	0.94	0.80	−0.25	0.52	0.34	0.30	0.25
		$\pi_{SS}^{o}$	−0.21	0.52	0.53	0.95	0.82	−0.25	0.52	0.28	0.37	0.25
												
	R	1	−0.01	0.83	0.58	1.00	0.98	−0.08	0.36	0.50	1.00	0.98
		*π_RDS_*	0.05	0.80	0.65	1.00	0.99	−0.04	0.31	0.59	1.00	0.99
		*π_SS_*	0.06	1.15	0.62	1.00	0.99	−0.06	0.32	0.49	1.00	0.99
		$\pi_{SS}^{u}$	0.05	1.11	0.60	1.00	0.99	−0.07	0.33	0.49	1.00	0.98
		$\pi_{SS}^{o}$	0.07	1.17	0.63	1.00	0.99	−0.06	0.31	0.51	1.00	0.99

Note: Clstr. is assumed at the seed level (S) and at the recruiter level (R). Clstr.: clustering; RB: relative bias; RMSE: root mean squared error; CI: coverage; NCI: neighbourhood bootstrap coverage; TCI: tree bootstrap coverage.

For linear regression, estimators are unbiased across all sampling fractions and network dependence parameters considered. The precision minimally increases with increasing sample fractions, but decreases with increasing network dependence. The coverage of the 95% confidence interval is consistently close to the nominal value; the unweighted estimator offers better coverage than weighted estimators.

For Poisson regression, estimators exhibit small biases across all sample fractions and network dependence; the unweighted estimator is slightly less biased than weighted estimators. The bias slightly increases with an increasing network dependence but does not consistently decrease with an increasing sample fraction. The estimator is less biased when clustering is assumed at the recruiter level. As in the linear case, the precision minimally increases with an increasing sample size, but does not consistently decrease with an increasing network dependence. The coverage of the 95% model-based confidence intervals are far below their nominal values; the coverage for the tree bootstrap confidence interval exceeds or is at the nominal value while, for the neighbourhood confidence interval, the coverage is slightly below or at the nominal value.

The logistic regression analysis yields estimators that are heavily biased across all sampling fractions, network dependence and sampling weights when clustering is assumed at the seed level. Models that assume clustering at the recruiter level yield estimators that exhibit small to negligible biases. The coverage of the model-based confidence intervals are below their nominal values; the coverage for the tree bootstrap confidence interval is above the nominal value, and the coverage for the neighbourhood bootstrap is slightly below or at the nominal value in most cases, when the bias is small to negligible.

These results are consistent with previous findings that omitting a non-confounding covariate (assuming the random effects model is correctly specified) does not induce bias for linear and Poisson regressions. In the logistic regression case, the omission of the covariate for the homophily effects induces attenuation bias because of the inappropriate collapsing of the contingency tables.^27,28^

To better understand the observed coverage for Poisson and logistic regressions, we reported the relative biases for the model-based and the bootstrap variance estimators in Web Supplement Tables S5 to S7. The model-based variance estimator underestimates uncertainty across all sampling fractions, levels of clustering and network dependence. The tree bootstrap variance estimator severely overestimates uncertainty in most cases while the neighbourhood bootstrap variance estimator is, in absolute value, less biased than both estimators in most cases, especially for the linear model. This aligns with previous findings in the RDS literature that, for the tree bootstrap method, covering at or above the nominal level generally comes at a significant cost in terms of power.^3,24^ Note that the widths of the model-based confidence intervals are smaller, while those of the tree bootstrap method are higher.

Model-based and bootstrap type I error rates were computed for all models using a continuous predictor from a normal distribution with mean 0 and standard deviation 1. The results, presented in Tables S8 to S10 of the Web Supplement, showed that model-based error rates for weighted models are consistently inflated, whereas neighbourhood and tree bootstrap error rates for weighted and unweighted (linear and logistic) models either match or are below the nominal rate. Note that model-based and neighbourhood bootstrap error rates for Poisson regression are slightly inflated across all models; tree bootstrap error rates are consistently below the nominal rate in this case.

### 4.4 Results for the second simulation study: Correlated predictor and degree

Table 2 report the results for the linear, Poisson and logistic regression. Weighted estimators are (slightly) less biased than unweighted estimators across all models, clustering levels and levels of correlation between the predictor and the degree. Furthermore, RDS-II weights perform as well as the SS weights across all models.

**Table 2.** Relative bias and root mean squared error of ${\hat{\beta }}_{1}$ , model-based CI, TCI and NCI for the 95% confidence interval of *β*_1_ with increasing association between predictor and degree (*ρ_d_*) and for various RDS weights (*π*).

		$\rho_{d}=0.4$					$\rho_{d}=0.6$				
Clstr.	*π*	RB	RMSE	CI	TCI	NCI	RB	RMSE	CI	TCI	NCI
**Linear regression**											
S	1	0	0.02	0.98	0.99	0.95	0	0.02	0.95	0.99	0.93
	*π _RDS_*	0	0.02	0.84	0.99	0.91	0	0.02	0.89	0.97	0.90
	*π _SS_*	0	0.02	0.85	0.99	0.91	0	0.02	0.90	0.97	0.91
	$\pi_{SS}^{u}$	0	0.02	0.85	0.99	0.91	0	0.02	0.90	0.98	0.91
	$\pi_{SS}^{o}$	0	0.02	0.85	0.99	0.91	0	0.02	0.90	0.97	0.91
											
R	1	0	0.01	0.96	0.99	0.95	0	0.02	0.95	0.99	0.92
	*π_RDS_*	0	0.02	0.88	0.99	0.93	0	0.02	0.86	0.99	0.91
	*π_SS_*	0	0.02	0.90	0.99	0.93	0	0.02	0.87	0.99	0.92
	$\pi_{SS}^{u}$	0	0.02	0.92	0.99	0.93	0	0.02	0.88	0.99	0.91
	$\pi_{SS}^{o}$	0	0.02	0.89	0.99	0.93	0	0.02	0.87	0.99	0.91
**Poisson regression**											
S	1	−0.02	1.01	0.32	0.94	0.92	−0.01	1.26	0.29	0.97	0.93
	*π _RDS_*	−0.01	0.82	0.33	0.96	0.92	0	1.02	0.32	0.96	0.92
	*π _SS_*	−0.01	0.83	0.32	0.96	0.92	0	1.06	0.31	0.96	0.92
	$\pi_{SS}^{u}$	−0.01	0.87	0.32	0.95	0.92	−0.01	1.11	0.31	0.96	0.92
	$\pi_{SS}^{o}$	−0.01	0.85	0.32	0.96	0.92	0	1.05	0.32	0.96	0.92
											
R	1	−0.05	2.17	0.35	0.98	0.96	−0.03	0.30	0.55	0.97	0.95
	*π_RDS_*	−0.02	1.04	0.38	0.98	0.95	−0.02	0.24	0.60	0.98	0.96
	*π_SS_*	−0.03	1.26	0.37	0.98	0.95	−0.02	0.25	0.60	0.98	0.96
	$\pi_{SS}^{u}$	−0.03	1.09	0.35	0.98	0.95	−0.02	0.26	0.60	0.98	0.96
	$\pi_{SS}^{o}$	−0.04	1.59	0.37	0.98	0.95	−0.02	0.25	0.60	0.98	0.96
**Logistic regression**											
S	1	−0.14	0.39	0.82	0.95	0.80	−0.14	0.37	0.79	0.94	0.82
	*π_RDS_*	−0.11	0.41	0.84	0.99	0.88	−0.10	0.37	0.79	0.97	0.91
	*π_SS_*	−0.11	0.40	0.83	0.99	0.87	−0.11	0.37	0.76	0.97	0.90
	$\pi_{SS}^{u}$	−0.12	0.39	0.84	0.99	0.86	−0.11	0.37	0.76	0.97	0.89
	$\pi_{SS}^{o}$	−0.11	0.40	0.85	0.99	0.87	−0.11	0.37	0.78	0.97	0.91
											
R	1	−0.05	0.52	0.46	1.00	0.98	−0.05	0.39	0.88	1.00	0.98
	*π_RDS_*	0.04	0.54	0.62	1.00	0.99	0.04	0.44	0.87	1.00	0.98
	*π_SS_*	0.03	0.52	0.60	1.00	0.98	0.03	0.43	0.88	1.00	0.98
	$\pi_{SS}^{u}$	0.01	0.50	0.54	1.00	0.98	0.02	0.43	0.86	1.00	0.98
	$\pi_{SS}^{o}$	0.03	0.52	0.61	1.00	0.98	0.03	0.43	0.87	1.00	0.98

Note: Clstr. is assumed at the seed level (S) and at the recruiter level (R). Clstr.: clustering; RB: relative bias; RMSE: root mean squared error; CI: coverage; NCI: neighbourhood bootstrap coverage; TCI: tree bootstrap coverage.

### 4.5 Summary and guidelines

Our results show that ignoring homophily-driven effects, if present, induces a negligible to small bias for linear and Poisson models while, for logistic regression, this strategy induces a substantial bias in the estimates when clustering is assumed at the seed level, and less bias but increased variability when clustering is assumed at the recruiter level. Moreover, misspecifying the SAR correlation model for the random effects induces an increasing bias as the dependence within the network increases, as well as a poor coverage of the model-based confidence interval for Poisson and logistic regressions. Bootstrap-based confidence intervals yield better coverage than model-based confidence intervals, particularly for Poisson and logistic regressions. Also, fitting mixed models in which clustering is assumed at the recruiter level yields estimators with less bias than models in which clustering is assumed at the seed level.

As for RDS weights, unweighted regression methods consistently outperform weighted methods in terms of precision and coverage when the predictor is uncorrelated with degree at the population level. Furthermore, the model-based type I error rate for unweighted models consistently matches the nominal rate while the bootstrap error rate for both unweighted and weighted (linear and logistic) models either match or are below the nominal error rate. The difference in precision can be attributed to the diffusion of the degree distribution of the network, which resulted in individuals having more small and large weights than expected by chance, hence increased variability in the estimators.^29^ On the other hand, weighted regression methods consistently outperform unweighted methods in terms of bias and precision when the predictor is correlated with degree.

We can therefore provide some general guidance for regression in RDS studies: (i) analyses that omit homophily-driven effects terms, while including a random effect for recruiter, outperform other modelling strategies in terms of bias, and (ii) weighted regression methods outperform unweighted regression methods in terms of bias and precision when the predictor is correlated with degree; when the predictor is uncorrelated with degree, weighting the model only increases variability in the estimates. Model-based type I error rates are high for weighted regression while bootstrap error rates either match or are below the nominal rate for both weighted and unweighted regressions. As observed previously,^24^ neighbourhood bootstrap provides better estimators of standard errors than any existing alternatives in all simulation scenarios.

## 5 Case study

We now turn to an analysis of the Engage study, a Canadian study conducted in three cities: Montreal, Toronto and Vancouver. The study aims to determine the individual, social and community-level risk factors for transmission of HIV and sexually transmitted infections and related behaviours within the GBM community. In this example, we focus on the data collected in Montreal. The Engage data-analysis team designed two databases and a tracker to monitor the RDS recruitment process. The study led to the recruitment of *n *=* *1179 GBM from Montreal between February 2017 through June 2018. Approximately 45% of recruited individuals were successful at recruiting, and 82% of these effective recruiters brought one to three peers into the study; 6 seeds of a total of 27 seed participants were unsuccessful at starting recruitment chains.

### 5.1 Descriptive statistics

Treatment optimism was measured on a scale of 12 items.^30^ All items were measured on a 4-point Likert scale (strongly disagree, disagree, agree and strongly agree). The optimism score (TMTOPT) was obtained by summing 10 items and subtracting 2 items. This gives a range of possible values between 0 (highly skeptical) and 36 (highly optimistic).

Age, education and income were found to be correlates of optimism through a range of bivariate analyses;^8^ we chose these same socio-demographic characteristics, among others, as possible predictors for treatment optimism. Descriptive (unweighted) statistics for these variables in the sample are presented in Table S11 of the Web Supplement. Around 33% of respondents were aged less than 30, about 70% were born in Canada, less than a third had a high school diploma or lower, and about 58% earned less than $30,000. Younger and more educated participants are less optimistic with regard to HIV treatment than other socio-demographic groups; the absolute difference is more pronounced for age. Furthermore, participants who were born in Canada and those who earn less than $30,000 in annual income have higher optimism scores than other participants.

### 5.2 Model fitting

We chose the potential socio-demographic characteristics correlates as predictors of HIV optimism for the aforementioned reasons. We fit various linear mixed-effects models with seed-specific and recruiter-specific random intercepts, in a weighted and unweighted fashion, for comparison purposes. Parameter estimates, standard errors and 95% (model-based and bootstrap) confidence intervals are reported in Table 3.

**Table 3.** Point estimates, standard errors and asymptotic 95% confidence intervals for a linear mixed model applied to the Engage Montreal data, where clustering is assumed at the seed level (S) and at the recruiter level (R), estimated without weights (1), with RDS-II (*π_RDS_*) weights and SS weights (*π_SS_*).

		S			R		
*π*		Est.	SE	CI	Est.	SE	CI
1	Constant	16.21	0.48	[15.3, 17.1]	16.21	0.45	[15.3, 17.1]
	Age (≤30)	−0.72	0.40	[−1.5, 0.0]	−0.72	0.44	[−1.6, 0.1]
	Education (< college)	0.92	0.90	[−0.8, 2.7]	0.85	1.00	[−1.1, 2.8]
	Born in Canada	0.58	0.43	[−0.3, 1.4]	0.55	0.49	[−0.4, 1.5]
	Annual income (≤$30,000)	0.38	0.41	[−0.4, 1.2]	0.43	0.43	[−0.4, 1.3]
	Born in Canada llege) rrel	−1.96	1.04	[−4.0, 0.0]	−1.93	1.15	[−4.2, 0.3]
	*σ*_0_(*ρ*)	0.0 (0.0)	–	–	1.33 (0.06)	–	–
							
*π_RDS_*	Constant	15.09	0.60	[13.9, 16.3]	15.50	0.66	[14.2, 16.8]
	Age (≤30)	−0.84	0.59	[−2.0, 0.3]	−0.86	0.61	[−2.1, 0.3]
	Education (< college)	2.62	1.42	[−0.2, 5.4]	0.59	1.17	[−1.7, 2.9]
	Born in Canada	0.93	0.63	[−0.3, 2.2]	0.56	0.82	[−1.0, 2.2]
	Annual income (≤$30,000)	1.30	0.69	[−0.1, 2.6]	1.54	0.62	[0.3, 2.8]
	Born in Canada llege) rrel	−4.34	1.75	[−7.8, −0.9]	−2.53	1.60	[−5.7, 0.6]
	*σ*_0_(*ρ*)	0.96 (0.01)	–	–	3.12 (0.15)	–	–
							
*π_SS_*	Constant	15.09	0.60	[13.9, 16.3]	15.51	0.65	[14.2, 16.8]
	Age (≤30)	−0.84	0.59	[−2.0, 0.3]	−0.86	0.60	[−2.0, 0.3]
	Education (< college)	2.62	1.41	[−0.2, 5.4]	0.63	1.16	[−1.6, 2.9]
	Born in Canada	0.93	0.62	[−0.3, 2.1]	0.57	0.80	[−1.0, 2.1]
	Annual income (≤ $30,000)	1.29	0.68	[0, 2.6]	1.52	0.61	[0.3, 2.7]
	Born in Canada llege) rrel	−4.32	1.74	[−7.7, −0.9]	−2.55	1.58	[−5.6, 0.5]
	*σ*_0_(*ρ*)	0.95 (0.0)	–	–	3.10 (0.02)	–	–

Note: The standard deviation of the random intercept is *σ*_0_ and the intraclass correlation is *ρ*. SE: standard error; CI: confidence interval.

We performed non-parametric Mann-Whitney U-tests to compare the distribution of degree between groups defined by the socio-demographic characteristics. The null hypothesis of the test is that for randomly selected values of degrees *d_i_* and *d_j_* from two groups, the probability of *d_i_* being greater than *d_j_* is equal to the probability of *d_j_* being greater than *d_i_*. In the Engage sample, the p-values of the test for age, education, being born in Canada and annual income are <0.01, 0.13, <0.01 and <0.01, respectively. This suggests differences in the median number of social connections between groups defined by age, being born in Canada and the annual income of participants, thus suggesting the use of weighted regression.

Guided by the simulations presented in Section 4.2 and by the discussion in the preceding paragraph, we focus on the weighted regression estimates with clustering at the recruiter level. We computed standard error estimates and 95% confidence intervals using the neighbourhood bootstrap method. The results show that annual income is significantly (and positively) associated with the optimism about the efficacy of the treatment, with a change of 1.5 points in the expected optimism score.

It is also worth noting that the directions of the associations between each covariate and the optimism score are consistent across all levels of clustering, regardless of the chosen RDS weight. However, the conclusions in terms of significance of the parameter effects differ whether we fit models with seed-specific random effects or recruiter-specific random effects.

We performed non-parametric hypothesis tests to decide whether or not to weight the model. It is important to highlight that we have not evaluated this approach, but rather use it as an informal tool to guide our analyses. Reasonably, a non-significant test does not exclude the possibility that there may be differences in the degree distribution across levels defined by the predictor, suggesting at least the use of weighted regression as a sensitivity check.

In our analyses, we chose socio-demographic factors as potential predictors of treatment optimism based on available evidence in the literature, but we have not tried to fully understand all predictors of the treatment score construct. Thus, this is a limited consideration of all potential predictors of treatment optimism, which can be further extended as more associational studies are conducted on the subject.

## 6 Discussion

The development of regression methods for RDS is limited by a missing data problem as the observed RDS data reveal only partial information about the structure of the population network. To our knowledge, this paper is the first to frame regression modelling for RDS as a missing data problem for which the partially observed network has serious implications on the validity of inference. Furthermore, we have provided additional investigations into the open question of the use of these design weights in regression settings, where the well-known results for RDS-II and SS weights do not automatically translate. Finally, we performed the first assessment of the tree bootstrap in a regression setting, and compared its performance to the novel neighbourhood bootstrap method.

We proposed alternative modelling strategies for RDS when the network is partially missing. Our results showed that ignoring homophily-driven effects, if present, induces a small to negligible bias in the parameter estimator (of the homophilic covariate) for linear and Poisson models while inducing a substantial bias for logistic regression when clustering is assumed at the seed level. Furthermore, misspecifying the correlation model induces an increasing bias as the dependence within the RDS network increases, and poor coverage for the model-based confidence intervals. In this case, the neighbourhood bootstrap method yields a variance estimator that is less biased than the model-based and the tree bootstrap variance estimators while offering confidence intervals with coverages that are slightly below or at the nominal level for linear and Poisson regression. We also showed that weighted regression methods outperform unweighted regression methods in terms of bias when the predictor is correlated with degree, assuming that there is no missing covariate in the model. Weighting the model only adds variability in the estimates when predictor and outcome are uncorrelated. Model-based type I error rates for weighted regression methods are highly inflated, while bootstrap error rates either match or are below the nominal rate for weighted and unweighted regressions.

In the case study, we restricted our analyses to the Engage Montreal dataset. This could be extended to the analysis of the data collected in Toronto and Vancouver by pooling across cities. This problem of conducting regression analyses using multi-city/state RDS data can be easily embedded within our inferential framework, if we can assume that city-specific networks are drawn from the same population network. This will be the subject of future work.

## Supplemental Material

sj-pdf-1-smm-10.1177_09622802211032713 - Supplemental material for General regression methods for respondent-driven sampling data Supplemental material, sj-pdf-1-smm-10.1177_09622802211032713 for General regression methods for respondent-driven sampling data by Mamadou Yauck, Erica EM Moodie, Herak Apelian, Alain Fourmigue, Daniel Grace, Trevor Hart, Gilles Lambert and Joseph Cox in Statistical Methods in Medical Research
