# Supplementary material for: General regression methods for respondent-driven sampling data
Source: Stat Methods Med Res. 2021 Jul 28;30(9):2105–18. doi: 10.1177/09622802211032713 (PMC8424528; doi:10.1177/09622802211032713)
Supplement: sj-pdf-1-smm-10.1177_09622802211032713 - Supplemental material for General regression methods for respondent-driven sampling data [file sj-pdf-1-smm-10.1177_09622802211032713.pdf]

Supporting information for General Regression Methods for Respondent-Driven Sampling Data

Mamadou Yauck*[1], Erica E. M. Moodie[1], Herak Apelian[1], Alain Fourmigue[1], Daniel Grace[3], Trevor A. Hart[4], Gilles Lambert[2], and Joseph Cox[1]

[1]McGill University, Montreal, Québec, Canada
[2]Institut national de santé publique du Québec, Montreal, Québec, Canada
[3]Dalla Lana School of Public Health, University of Toronto
[4] Ryerson University, Toronto, Ontario, Canada

This document gives additional results from the simulation study presented in Section 4 and the case study presented in Section 5 of the manuscript. In Web Appendix A1, we reported the relative bias and the root mean squared error of $\hat{\beta}_1$, and the coverage of the 95% confidence interval of β_1 for a sample fraction of $f = 10\%$. We report, in Web Appendix A2, the performance of the MLE of β_1 when the fitted model includes correlated predictors of the outcome. In Web Appendix B, we reported the relative biases for the model-based, the tree bootstrap and the neighbourhood bootstrap variance estimators in Tables S5, S6 and S7, respectively. We reported type I error rates in Tables S8-S10 of Web Appendix C. In Web Appendix D, we reported descriptive statistics of the socio-demographic characteristics in an RDS sample of gay, bisexual and other men who have sex with men, collected in Montreal via the Engage study.

*E-mail: *mamadou.yauck@mcgill.ca*

Web Appendix A1: Relative bias and root mean squared error of $\hat{\beta}_1$, and coverage of the 95% confidence interval of β_1 for a sample fraction $f = 10\%$

Table S1: Linear - Relative bias and root mean squared error of $\hat{\beta}_1$, model-based coverage (CI), the tree bootstrap coverage (TCI) and the neighbourhood bootstrap coverage (NCI) of the 95% confidence interval of β_1 for increasing levels of network dependence (ρ) and various RDS weights (π). Clustering (Clstr.) is assumed at the seed level (S) and at the recruiter level (R).

ρ	Clstr.	π	RB	RMSE	CI	TCI	NCI
0.05	S	1	0	0.11	0.94	0.99	0.94
		π_{RDS}	0	0.15	0.90	0.98	0.94
		π_{SS}	0	0.14	0.92	0.98	0.94
		π_{SS}^u	0	0.14	0.92	0.98	0.93
		π_{SS}^o	0	0.15	0.92	0.98	0.93
	R	1	0	0.11	0.91	0.99	0.95
		π_{RDS}	0	0.15	0.88	0.99	0.92
		π_{SS}	0	0.14	0.89	0.99	0.93
		π_{SS}^u	0	0.14	0.88	0.99	0.93
		π_{SS}^o	0	0.14	0.88	0.99	0.92
0.1	S	1	0	0.15	0.96	1.00	0.93
		π_{RDS}	0	0.15	0.91	0.98	0.94
		π_{SS}	0	0.15	0.94	0.99	0.93
		π_{SS}^u	0	0.15	0.94	0.99	0.93
		π_{SS}^o	0	0.15	0.93	0.99	0.94
	R	1	0	0.13	0.91	1.00	0.93
		π_{RDS}	0	0.16	0.90	1.00	0.92
		π_{SS}	0	0.15	0.89	1.00	0.92
		π_{SS}^u	0	0.15	0.90	1.00	0.92
		π_{SS}^o	0	0.15	0.90	1.00	0.92

Table S2: Poisson - Relative bias and root mean squared error of $\hat{\beta}_1$, model-based coverage (CI), the tree bootstrap coverage (TCI) and the neighbourhood bootstrap coverage (NCI) of the 95% confidence interval of β_1 for increasing levels of network dependence (ρ) and various RDS weights (π). Clustering (Clstr.) is assumed at the seed level (S) and at the recruiter level (R).

ρ	Clstr.	π	RB	RMSE	CI	TCI	NCI
0.05	S	1	-0.05	0.51	0.46	0.98	0.89
		π_{RDS}	-0.07	0.54	0.42	0.98	0.90
		π_{SS}	-0.07	0.54	0.41	0.98	0.88
		π_{SS}^u	-0.07	0.54	0.42	0.98	0.88
		π_{SS}^o	-0.07	0.54	0.41	0.98	0.88
	R	1	-0.06	0.45	0.54	0.97	0.92
		π_{RDS}	-0.06	0.50	0.53	0.96	0.91
		π_{SS}	-0.08	0.57	0.52	0.96	0.91
		π_{SS}^u	-0.07	0.50	0.52	0.96	0.91
		π_{SS}^o	-0.08	0.59	0.52	0.96	0.91
0.1	S	1	-0.07	0.53	0.48	0.96	0.92
		π_{RDS}	-0.08	0.52	0.43	0.94	0.90
		π_{SS}	-0.08	0.52	0.45	0.94	0.90
		π_{SS}^u	-0.08	0.52	0.45	0.94	0.90
		π_{SS}^o	-0.08	0.52	0.45	0.94	0.90
	R	1	-0.10	0.55	0.51	0.98	0.91
		π_{RDS}	-0.09	0.54	0.55	0.97	0.90
		π_{SS}	-0.11	0.54	0.56	0.96	0.89
		π_{SS}^u	-0.12	0.54	0.56	0.96	0.89
		π_{SS}^o	-0.12	0.55	0.56	0.96	0.89

Table S3: Logistic - Relative bias and root mean squared error of $\hat{\beta}_1$, model-based coverage (CI), the tree bootstrap coverage (TCI) and the neighbourhood bootstrap coverage (NCI) of the 95% confidence interval of β_1 for increasing levels of network dependence (ρ) and various RDS weights (π). Clustering (Clstr.) is assumed at the seed level (S) and at the recruiter level (R).

ρ	Clstr.	π	RB	RMSE	CI	TCI	NCI
0.05	S	1	-0.14	0.50	0.84	0.94	0.89
		π_{RDS}	-0.12	0.57	0.70	0.93	0.90
		π_{SS}	-0.12	0.56	0.72	0.93	0.89
		π^u_{SS}	-0.12	0.56	0.72	0.93	0.89
		π^o_{SS}	-0.12	0.57	0.72	0.93	0.90
	R	1	0.10	1.43	0.68	0.99	0.98
		π_{RDS}	0.12	1.52	0.62	0.99	0.99
		π_{SS}	0.12	1.51	0.64	0.99	0.98
		π^u_{SS}	0.12	1.51	0.64	0.99	0.98
		π^o_{SS}	0.12	1.51	0.64	0.99	0.98
0.1	S	1	-0.21	0.56	0.62	0.88	0.76
		π_{RDS}	-0.17	0.55	0.58	0.92	0.88
		π_{SS}	-0.17	0.55	0.57	0.93	0.87
		π^u_{SS}	-0.18	0.55	0.57	0.93	0.87
		π^o_{SS}	-0.17	0.55	0.57	0.93	0.87
	R	1	1.39	0.08	0.62	1.00	0.98
		π_{RDS}	0.10	1.42	0.58	1.00	0.99
		π_{SS}	0.10	1.45	0.61	1.00	0.99
		π^u_{SS}	0.10	1.45	0.61	1.00	0.99
		π^o_{SS}	0.10	1.45	0.61	1.00	0.99

Web Appendix A2: Relative bias and root mean squared error of $\hat{\beta}_1$, and coverage of the 95% confidence interval of β_1 in the presence of correlated predictors.

Table S4: Relative bias and root mean squared error of $\hat{\beta}_1$, model-based coverage (CI) of the 95% confidence interval of β_1 for various RDS weights (π). Clustering (Clstr.) is assumed at the seed level (S) and at the recruiter level (R).

Clstr.	π	RB	RMSE	CI
		**Linear**	**Regression**	
S	1	0	0.08	0.94
	π_{RDS}	0	0.12	0.92
	π_{SS}	0	0.11	0.90
R	1	0	0.09	0.92
	π_{RDS}	0	0.11	0.87
	π_{SS}	0	0.11	0.88
		**Poisson**	**Regression**	
S	1	-0.10	0.43	0.38
	π_{RDS}	-0.10	0.44	0.37
	π_{SS}	-0.10	0.44	0.37
R	1	-0.07	0.47	0.35
	π_{RDS}	-0.08	0.46	0.39
	π_{SS}	-0.11	0.48	0.39
		**Logistic**	**Regression**	
S	1	-0.22	0.50	0.54
	π_{RDS}	-0.18	0.48	0.53
	π_{SS}	-0.19	0.49	0.53
R	1	-0.03	0.64	0.60
	π_{RDS}	0.04	0.63	0.58
	π_{SS}	0.05	1.19	0.58

Web Appendix B: Bias of standard errors estimators for the linear, Poisson and logistic models

Table S5: Linear - Relative biases for the model-based variance estimator (V), the tree bootstrap estimator (V_B) and the neighbourhood bootstrap estimator (V_N) of $\hat{\beta}_1$ for an increasing sample fraction (f), network dependence (ρ) and various RDS weights (π). Confidence intervals widths (W) are displayed in parentheses.

			$f = 20\%$			$f = 80\%$		
ρ	Clstr.	π	V (W)	V_B (W)	V_N (W)	V (W)	V_B (W)	V_N (W)
0.05	S	1	-0.18 (0.21)	0.85 (0.37)	-0.04 (0.23)	-0.17 (0.11)	>1 (>0.17)	-0.14 (0.11)
		π_{RDS}	-0.27 (0.23)	0.96 (0.38)	-0.08 (0.26)	-0.33 (0.16)	>1 (>0.28)	-0.16 (0.18)
		π_{SS}	-0.24 (0.24)	0.95 (0.38)	-0.07 (0.26)	-0.15 (0.14)	>1 (>0.22)	-0.16 (0.14)
		π_{SS}^u	-0.23 (0.24)	0.94 (0.38)	-0.06 (0.27)	-0.15 (0.14)	>1 (>0.22)	-0.15 (0.14)
		π_{SS}^o	-0.25 (0.24)	0.94 (0.38)	-0.07 (0.26)	-0.16 (0.14)	>1 (>0.22)	-0.16 (0.14)
	R	1	-0.09 (0.26)	>1 (>0.40)	0.08 (0.28)	-0.21 (0.10)	>1 (>0.17)	0.07 (0.12)
		π_{RDS}	-0.28 (0.27)	>1 (>0.44)	0.04 (0.32)	-0.20 (0.17)	>1 (>0.28)	0.03 (0.20)
		π_{SS}	-0.25 (0.24)	>1 (>0.40)	0.05 (0.28)	-0.15 (0.14)	>1 (>0.22)	0.06 (0.16)
		π_{SS}^u	-0.22 (0.24)	>1 (>0.40)	0.07 (0.28)	-0.16 (0.14)	>1 (>0.22)	0.06 (0.16)
		π_{SS}^o	-0.26 (0.27)	>1 (>0.44)	0.05 (0.32)	-0.14 (0.14)	>1 (>0.22)	0.05 (0.16)
0.1	S	1	0.05 (0.28)	>1 (>0.40)	-0.06 (0.27)	-0.21 (0.14)	>1 (>0.22)	-0.02 (0.15)
		π_{RDS}	-0.28 (0.30)	>1 (>0.50)	-0.09 (0.34)	-0.39 (0.18)	>1 (>0.33)	-0.02 (0.23)
		π_{SS}	-0.23 (0.28)	>1 (>0.44)	-0.08 (0.30)	-0.22 (0.14)	>1 (>0.22)	-0.03 (0.15)
		π_{SS}^u	-0.19 (0.28)	>1 (>0.44)	-0.08 (0.30)	-0.21 (0.14)	>1 (>0.22)	-0.02 (0.15)
		π_{SS}^o	-0.25 (0.27)	>1 (>0.44)	-0.09 (0.30)	-0.23 (0.14)	>1 (>0.22)	-0.03 (0.15)
	R	1	-0.06 (0.30)	>1 (>0.44)	0.08 (0.33)	-0.28 (0.13)	>1 (>0.22)	0.12 (0.16)
		π_{RDS}	-0.23 (0.31)	>1 (>0.50)	0 (0.35)	-0.35 (0.19)	>1 (>0.33)	0.11 (0.25)
		π_{SS}	-0.19 (0.32)	>1 (>0.50)	0.02 (0.36)	-0.29 (0.13)	>1 (>0.22)	0.10 (0.16)
		π_{SS}^u	-0.16 (0.32)	>1 (>0.50)	0.02 (0.36)	-0.29 (0.13)	>1 (>0.22)	0.11 (0.16)
		π_{SS}^o	-0.20 (0.31)	>1 (>0.50)	0.01 (0.35)	-0.29 (0.13)	>1 (>0.22)	0.10 (0.16)

Table S6: Poisson - Relative biases for the model-based variance estimator (V), the tree bootstrap estimator (V_B) and the neighbourhood bootstrap estimator (V_N) of $\hat{\beta}_1$ for an increasing sample fraction (f), network dependence (ρ) and various RDS weights (π). Confidence intervals widths (W) are displayed in parentheses.

			$f = 20\%$			$f = 80\%$		
ρ	Clstr.	π	V (W)	V_B (W)	V_N (W)	V (W)	V_B (W)	V_N (W)
0.05	S	1	-0.93 (0.42)	0.71 (2.10)	-0.19 (1.45)	-0.95 (0.26)	0.27 (1.32)	-0.48 (0.85)
		π_{RDS}	-0.93 (0.51)	0.86 (2.62)	-0.12 (1.80)	-0.97 (0.26)	0.24 (1.66)	-0.56 (0.99)
		π_{SS}	-0.93 (0.50)	0.81 (2.53)	-0.14 (1.74)	-0.97 (0.23)	0.29 (1.51)	-0.54 (0.90)
		π_{SS}^u	-0.93 (0.48)	0.76 (2.39)	-0.16 (1.65)	-0.96 (0.26)	0.23 (1.43)	-0.52 (0.90)
		π_{SS}^o	-0.93 (0.50)	0.83 (2.54)	-0.13 (1.75)	-0.97 (0.23)	0.26 (1.50)	-0.54 (0.90)
	R	1	-0.90 (0.45)	>1 (>1.99)	0.54 (1.75)	-0.96 (0.19)	>1 (>1.33)	0.50 (1.15)
		π_{RDS}	-0.94 (0.44)	>1 (>2.55)	0.57 (2.26)	-0.98 (0.17)	>1 (>1.66)	0.46 (1.42)
		π_{SS}	-0.94 (0.45)	>1 (>2.60)	0.44 (2.21)	-0.97 (0.18)	>1 (>1.50)	0.43 (1.26)
		π_{SS}^u	-0.93 (0.47)	>1 (>2.49)	0.53 (2.18)	-0.97 (0.18)	>1 (>1.50)	0.48 (1.29)
		π_{SS}^o	-0.94 (0.45)	>1 (>2.60)	0.53 (2.28)	-0.97 (0.19)	>1 (>1.55)	0.45 (1.32)
0.1	S	1	-0.81 (0.73)	0.56 (2.10)	-0.34 (1.37)	-0.95 (0.31)	0.27 (1.55)	-0.61 (0.86)
		π_{RDS}	-0.86 (0.64)	0.56 (2.15)	-0.26 (1.48)	-0.97 (0.27)	0.28 (1.77)	-0.60 (0.99)
		π_{SS}	-0.87 (0.62)	0.56 (2.15)	-0.27 (1.47)	-0.96 (0.30)	0.26 (1.63)	-0.62 (0.89)
		π_{SS}^u	-0.88 (0.60)	0.55 (2.15)	-0.29 (1.45)	-0.96 (0.30)	0.34 (1.68)	-0.62 (0.89)
		π_{SS}^o	-0.87 (0.60)	0.61 (2.19)	-0.27 (1.47)	-0.97 (0.26)	0.27 (1.68)	-0.62 (0.92)
	R	1	-0.93 (0.54)	>1 (>2.88)	0.57 (2.55)	-0.97 (0.21)	>1 (>1.72)	0.54 (1.51)
		π_{RDS}	-0.93 (0.53)	>1 (>2.83)	0.57 (2.50)	-0.98 (0.19)	>1 (>1.88)	0.50 (1.63)
		π_{SS}	-0.96 (0.49)	>1 (>3.49)	0.49 (3.01)	-0.97 (0.22)	>1 (>1.83)	0.48 (1.57)
		π_{SS}^u	-0.95 (0.52)	>1 (>3.33)	0.47 (2.85)	-0.97 (0.22)	>1 (>1.83)	0.49 (1.58)
		π_{SS}^o	-0.96 (0.49)	>1 (>3.49)	0.55 (3.07)	-0.97 (0.23)	>1 (>1.88)	0.57 (1.67)

Table S7: Logistic - Relative biases for the model-based variance estimator (V), the tree bootstrap estimator (V_B) and the neighbourhood bootstrap estimator (V_N) of $\hat{\beta}_1$ for an increasing sample fraction (f), network dependence (ρ) and various RDS weights (π). Confidence intervals widths (W) are displayed in parentheses.

			$f = 20\%$			$f = 80\%$		
ρ	Clstr.	π	V (W)	V_B (W)	V_N (W)	V (W)	V_B (W)	V_N (W)
0.05	S	1	-0.68 (0.95)	>1 (>2.38)	-0.46 (1.24)	-0.93 (0.44)	-0.71 (0.91)	-0.92 (0.48)
		π_{RDS}	-0.70 (1.00)	>1 (>2.60)	-0.21 (1.64)	-0.95 (0.43)	-0.62 (1.18)	-0.89 (0.64)
		π_{SS}	-0.70 (0.99)	>1 (>2.55)	-0.26 (1.55)	-0.94 (0.44)	-0.71 (0.97)	-0.92 (0.51)
		π_{SS}^u	-0.70 (0.99)	>1 (>2.55)	-0.29 (1.52)	-0.94 (0.43)	-0.72 (0.93)	-0.92 (0.50)
		π_{SS}^o	-0.70 (1.00)	>1 (>2.60)	-0.24 (1.60)	-0.94 (0.45)	-0.70 (1.00)	-0.92 (0.52)
	R	1	-0.91 (1.59)	>1 (>7.48)	>1 (>7.48)	-0.91 (0.50)	>1 (>2.38)	>1 (>2.38)
		π_{RDS}	-0.97 (0.81)	>1 (>6.60)	>1 (>6.60)	-0.97 (0.24)	>1 (>1.99)	>1 (>1.99)
		π_{SS}	-0.97 (0.81)	>1 (>6.65)	>1 (>6.65)	-0.90 (0.48)	>1 (>2.16)	>1 (>2.16)
		π_{SS}^u	-0.97 (0.86)	>1 (>7.04)	>1 (>7.04)	-0.90 (0.49)	>1 (>2.22)	>1 (>2.22)
		π_{SS}^o	-0.97 (0.81)	>1 (>6.60)	>1 (>6.60)	-0.89 (0.49)	>1 (>2.11)	>1 (>2.11)
0.1	S	1	-0.80 (0.93)	>1 (>2.94)	-0.55 (1.39)	-0.96 (0.40)	-0.84 (0.80)	-0.94 (0.49)
		π_{RDS}	-0.77 (0.98)	>1 (>2.88)	-0.56 (1.35)	-0.96 (0.41)	-0.76 (1.02)	-0.91 (0.62)
		π_{SS}	-0.78 (0.96)	>1 (>2.88)	-0.59 (1.30)	-0.96 (0.41)	-0.83 (0.84)	-0.94 (0.50)
		π_{SS}^u	-0.78 (0.96)	>1 (>2.88)	-0.62 (1.26)	-0.96 (0.41)	-0.84 (0.81)	-0.94 (0.50)
		π_{SS}^o	-0.78 (0.96)	>1 (>2.88)	-0.59 (1.30)	-0.96 (0.41)	-0.83 (0.84)	-0.94 (0.50)
	R	1	-0.84 (1.30)	>1 (>4.60)	>1 (>4.60)	-0.90 (0.45)	>1 (>1.99)	>1 (>1.99)
		π_{RDS}	-0.73 (1.63)	>1 (>4.43)	>1 (>4.43)	-0.87 (0.44)	>1 (>1.72)	>1 (>1.72)
		π_{SS}	-0.75 (2.25)	>1 (>6.37)	>1 (>6.37)	-0.88 (0.43)	>1 (>1.77)	>1 (>1.77)
		π_{SS}^u	-0.76 (2.13)	>1 (>6.15)	>1 (>6.15)	-0.89 (0.43)	>1 (>1.83)	>1 (>1.83)
		π_{SS}^o	-0.74 (2.34)	>1 (>6.49)	>1 (>6.49)	-0.88 (0.42)	>1 (>1.72)	>1 (>1.72)

Web Appendix C: Type I error rate

Table S8: Model-based type I error rate of linear (Lin), Poisson (Pois) and logistic (Log) regressions for an increasing sample fraction (f), network dependence (ρ) and various RDS weights (π).

			$f = 20\%$			$f = 80\%$		
ρ	Clstr.	π	Lin	Pois	Log	Lin	Pois	Log
0.05	S	1	0.05	0.15	0.05	0.05	0.14	0.05
		π_{RDS}	0.13	0.27	0.13	0.14	0.19	0.16
		π_{SS}	0.11	0.26	0.11	0.07	0.14	0.07
		π_{SS}^u	0.10	0.26	0.11	0.07	0.14	0.06
		π_{SS}^o	0.12	0.27	0.11	0.07	0.14	0.07
	R	1	0.04	0.11	0.04	0.03	0.08	0.06
		π_{RDS}	0.12	0.16	0.10	0.09	0.16	0.14
		π_{SS}	0.11	0.17	0.10	0.09	0.10	0.08
		π_{SS}^u	0.10	0.15	0.10	0.07	0.09	0.07
		π_{SS}^o	0.11	0.17	0.10	0.08	0.11	0.08
0.1	S	1	0.03	0.15	0.05	0.05	0.09	0.03
		π_{RDS}	0.10	0.16	0.09	0.13	0.20	0.06
		π_{SS}	0.09	0.16	0.10	0.06	0.12	0.05
		π_{SS}^u	0.09	0.16	0.09	0.06	0.12	0.05
		π_{SS}^o	0.08	0.16	0.10	0.07	0.12	0.05
	R	1	0.07	0.10	0.01	0.07	0.12	0.06
		π_{RDS}	0.15	0.13	0.07	0.14	0.18	0.15
		π_{SS}	0.15	0.14	0.06	0.08	0.12	0.07
		π_{SS}^u	0.14	0.13	0.05	0.08	0.12	0.06
		π_{SS}^o	0.15	0.14	0.07	0.08	0.13	0.08

Table S9: Neighbourhood bootstrap type I error rate of linear (Lin), Poisson (Pois) and logistic (Log) regressions for an increasing sample fraction (f), network dependence (ρ) and various RDS weights (π).

			$f = 20\%$			$f = 80\%$		
ρ	Clstr.	π	Lin	Pois	Log	Lin	Pois	Log
0.05	S	1	0.06	0.10	0.04	0.05	0.08	0.03
		π_{RDS}	0.09	0.11	0.06	0.07	0.10	0.06
		π_{SS}	0.08	0.11	0.06	0.06	0.09	0.05
		π_{SS}^u	0.08	0.10	0.05	0.06	0.09	0.05
		π_{SS}^o	0.08	0.11	0.06	0.06	0.09	0.05
	R	1	0.05	0.13	0.01	0.16	0.08	0.02
		π_{RDS}	0.06	0.11	0.01	0.06	0.09	0.01
		π_{SS}	0.05	0.10	0.01	0.05	0.08	0.01
		π_{SS}^u	0.05	0.10	0.01	0.05	0.08	0.02
		π_{SS}^o	0.06	0.08	0.01	0.05	0.08	0.01
0.1	S	1	0.04	0.11	0.05	0.04	0.08	0.04
		π_{RDS}	0.07	0.12	0.06	0.07	0.09	0.05
		π_{SS}	0.06	0.11	0.05	0.05	0.09	0.05
		π_{SS}^u	0.06	0.11	0.05	0.05	0.08	0.05
		π_{SS}^o	0.06	0.11	0.05	0.05	0.09	0.05
	R	1	0.06	0.11	0.01	0.04	0.09	0.02
		π_{RDS}	0.07	0.12	0.01	0.06	0.10	0.01
		π_{SS}	0.06	0.11	0.01	0.05	0.08	0.01
		π_{SS}^u	0.06	0.11	0.01	0.05	0.08	0.01
		π_{SS}^o	0.06	0.11	0.01	0.05	0.09	0.02

Table S10: Tree bootstrap type I error rate of linear (Lin), Poisson (Pois) and logistic (Log) regressions for an increasing sample fraction (f), network dependence (ρ) and various RDS weights (π).

			$f = 20\%$			$f = 80\%$		
ρ	Clstr.	π	Lin	Pois	Log	Lin	Pois	Log
0.05	S	1	0.02	0.04	0.01	0.01	0.02	0.01
		π_{RDS}	0.02	0.03	0.01	0.01	0.02	0.01
		π_{SS}	0.02	0.03	0.01	0.01	0.03	0.01
		π_{SS}^u	0.02	0.02	0.01	0.01	0.03	0.01
		π_{SS}^o	0.02	0.03	0.01	0.01	0.03	0.01
	R	1	0.01	0.03	0.01	0.01	0.02	0.01
		π_{RDS}	0.01	0.02	0.01	0.01	0.02	0.01
		π_{SS}	0.01	0.02	0.01	0.01	0.03	0.01
		π_{SS}^u	0.01	0.03	0.01	0.01	0.03	0.01
		π_{SS}^o	0.01	0.02	0.01	0.01	0.03	0.01
0.1	S	1	0.01	0.05	0.01	0.01	0.06	0.01
		π_{RDS}	0.02	0.03	0.01	0.01	0.02	0.01
		π_{SS}	0.02	0.03	0.01	0.01	0.02	0.01
		π_{SS}^u	0.02	0.04	0.01	0.01	0.03	0.01
		π_{SS}^o	0.02	0.03	0.01	0.01	0.02	0.01
	R	1	0.02	0.04	0.01	0.01	0.04	0.01
		π_{RDS}	0.02	0.03	0.01	0.01	0.03	0.01
		π_{SS}	0.02	0.02	0.01	0.01	0.02	0.01
		π_{SS}^u	0.02	0.03	0.01	0.01	0.02	0.01
		π_{SS}^o	0.02	0.03	0.01	0.01	0.02	0.01

Web Appendix D: Engage Montreal study - Descriptive statistics for the socio-demographic variables in the RDS sample

Table S11: Descriptive (unweighted) statistics of the RDS sample of n=1179 gay, bisexual and other men who have sex with men (GBM) recruits in Engage Montreal (2017-2018): number (n), percent (%) of socio-demographic characteristics and risk behaviors, mean (m) and standard deviation (SD) of the treatment optimism score (TMTOPT) broken drown by socio-demographic groups (reference group and others).

		**TMTOPT score**	
**Reference groups**		Ref.	Other
	n (%)	m (SD)	
**Socio-demographic characteristics**			
Age $\leq$ 30	384 (32.6)	16.2(5.2)	17.3(5.9)
Born in Canada	821 (69.6)	17.0(5.9)	16.7(5.4)
Highest diploma < college	352 (29.9)	16.7(6.0)	17.0(5.6)
$\leq$ 30 000$ in annual income	678 (57.5)	17.0(5.7)	16.8(5.8)
